# Closed microbial communities self-organize to persistently cycle carbon

**DOI:** 10.1073/pnas.2013564118

**Published:** 2021-11-05

**Authors:** Luis Miguel de Jesús Astacioa, Kaumudi H. Prabhakara, Zeqian Li, Harry Mickalide, Seppe Kuehn

**Affiliations:** ^a^Center for the Physics of Living Cells, University of Illinois at Urbana–Champaign, Urbana, IL 61801;; ^b^Department of Physics, University of Illinois at Urbana–Champaign, Urbana, IL 61801;; ^c^Center for the Physics of Evolving Systems, University of Chicago, Chicago, IL 60637;; ^d^Department of Ecology and Evolution, University of Chicago, Chicago, IL 60637

**Keywords:** microbial communities, carbon cycling, closed ecosystems, functional redundancy

## Abstract

Cycles of nutrients (N, P, etc.) and resources (C) are a defining emergent feature of ecosystems. Cycling plays a critical role in determining ecosystem structure at all scales, from microbial communities to the entire biosphere. Stable cycles are essential for ecosystem persistence because they allow resources and nutrients to be regenerated. Therefore, a central problem in ecology is understanding how ecosystems are organized to sustain robust cycles. Addressing this problem quantitatively has proved challenging because of the difficulties associated with manipulating ecosystem structure while measuring cycling. We address this problem using closed microbial ecosystems (CES), hermetically sealed microbial consortia provided with only light. We develop a technique for quantifying carbon cycling in hermetically sealed microbial communities and show that CES composed of an alga and diverse bacterial consortia self-organize to robustly cycle carbon for months. Comparing replicates of diverse CES, we find that carbon cycling does not depend strongly on the taxonomy of the bacteria present. Moreover, despite strong taxonomic differences, self-organized CES exhibit a conserved set of metabolic capabilities. Therefore, an emergent carbon cycle enforces metabolic but not taxonomic constraints on ecosystem organization. Our study helps establish closed microbial communities as model ecosystems to study emergent function and persistence in replicate systems while controlling community composition and the environment.

## Significance

Life on Earth depends on ecologically driven nutrient cycles to regenerate resources. Understanding how nutrient cycles emerge from a complex web of ecological processes is a central challenge in ecology. However, we lack model ecosystems that can be replicated, manipulated, and quantified in the laboratory, making it challenging to determine how changes in composition and the environment impact cycling. Enabled by a new high-precision method to quantify carbon cycling, we show that materially closed microbial ecosystems (CES) provided with only light self-organize to robustly cycle carbon. Studying replicate CES that support a carbon cycle reveals variable community composition but a conserved set of metabolic capabilities. Our study helps establish CES as model biospheres for studying how ecosystems persistently cycle nutrients.

Nutrient cycles are an important feature of ecosystems at all scales. The persistent cyclic flow of nutrients through ecosystems arises from a balance between complementary metabolic processes. How ecosystems are organized to facilitate this balance is an important question because cycling enables ecosystem persistence by continuously replenishing resources. As a result, global cycles of carbon (1) and nitrogen (2) are important organizing processes of life across the planet. On a smaller scale, microbial communities often exploit nutrient cycling to overcome local nutrient limitation from carbon fixation and respiration in microbial mats (3), to denitrification and nitrogen fixation in soils (4), sulfur oxidation and reduction in anaerobic marine microbial communities (5), and nutrient cycling in periphytic consortia (6).

The importance of nutrient cycling for ecosystems means that a key problem in ecology is understanding how the cyclic flow of nutrients emerges from interactions between organisms in communities (7). Microbial communities, owing to their small size, rapid replication rates, and tractability in the laboratory, are powerful model systems for discovering the principles governing ecosystem organization and function. For example, a conserved succession of bacteria with predictable metabolic capabilities describes the degradation of particulate organic carbon in marine microbial communities (8). Complex bacterial communities propagated in the laboratory reveal emergent cross-feeding between predictable taxa (9), and simple assembly rules govern the stable composition of synthetic communities (10).

However, few quantitative studies have exploited the advantages of microbial communities in the laboratory to uncover the principles governing the assembly of communities that cycle nutrients. A primary roadblock to studying nutrient cycling in model microbial communities is experimental: Most existing approaches use batch (9) or continuous culture (11), where nutrients are supplied externally at high rates. In these conditions, nutrient cycling rarely occurs since the external supply of nutrients favors those strains that can most rapidly exploit the supplied resource (8, 9). The continuous and rapid dilution of these systems means that slower-growing taxa are quickly washed out (12), frequently resulting in the assembly of communities with taxa that either exploit the primary resource or are sustained via strong mutualistic or commensal interactions (9, 13). Nutrient cycles occur when some nutrients are regenerated by the community itself, instead of being supplied exogenously.

Stable nutrient cycling therefore requires a balance between the production of byproducts (e.g., CO_2_ by respiration) and their consumption (CO_2_ fixation by photosynthesis) in a closed loop. For these reasons, cycling can arise in batch culture when growth rates are slow (14). Similarly, Winogradsky columns, where communities stratify along redox gradients from anoxic to oxic conditions, are important experimental tools that overcome the limitations of batch and chemostat methods to provide model systems for studying nutrient cycling (15–17). However, it remains a challenge to quantify nutrient cycling in these spatially structured communities. Here we seek to overcome some of the limitations of existing methods by establishing closed microbial ecosystems (CES) as model systems for understanding how communities are assembled to cycle nutrients. Therefore, we hope that CES can complement existing batch-culture, chemostat, and Winogradsky column-based approaches (18).

## Closed Microbial Communities

Several groups, including Obenhuber and Folsome (19) and Taub and McLaskey (20), have pioneered the use of CES as models for understanding the principles of emergent nutrient cycling. CES are milliliter-scale aquatic communities that are hermetically sealed and illuminated (19–23). Since no nutrients enter or leave a CES after assembly, persistence in these communities requires that nutrient cycles be sustained through photosynthesis. Complex CES have been shown to retain biological activity for decades in some cases (23). As such, CES are ideal model microbial ecosystems for understanding nutrient cycling (18). However, most work on CES to date has focused on applications to spaceflight (24) or population dynamics (22, 25, 26). Previous efforts to use CES to understand how communities cycle nutrients were limited by low-throughput measurements of cycling (20) and did not apply sequencing methods to quantify community structure.

Here we take a top–down approach (9, 19) to assemble replicate CES, comprising diverse bacterial consortia derived from soil and a domesticated algal species. We develop a high-precision method for quantifying carbon cycling in situ to show that our CES rapidly and persistently cycle carbon. We utilize 16S ribosomal RNA (rRNA) sequencing and metabolic profiling to reveal the conserved metabolic features of CES that cycle carbon.

## Carbon Cycling in Closed Microbial Communities

**Fig. 1:**
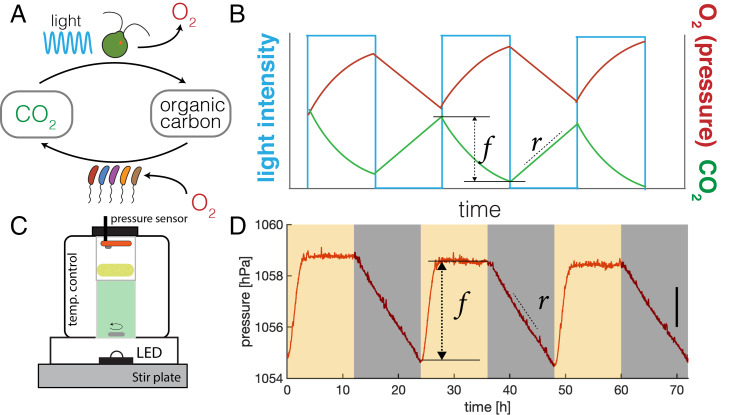
Quantifying carbon cycling in closed microbial ecosystems. (*A*) Schematic of carbon cycling in closed ecosystems. Cycling occurs via photosynthesis utilizing light to fix CO_2_ to organic carbon, producing O_2_ (top arrow), and respiration that utilizes O_2_ and organic carbon to produce CO_2_. (*B*) Sketch of changes in total O_2_ or pressure (red line) and CO_2_ (green line) in a CES subjected to cycles of light and dark (blue line). Sketch assumes photosynthetic rate exceeds respiration rate during the light phase. *r* is the rate of increase of CO_2_ during the dark phase. *f* is the net decrease in CO_2_ during the light phase. Assuming respiratory and photosynthetic quotients of one, O_2_ dynamics mirror CO_2_. Since O_2_ is 30-fold less soluble in water than CO_2_, changes in pressure quantify changes in O_2_ and CO_2_ concentrations in a CES (*SI Appendix*). (*C*) A schematic of our custom cultivation devices for quantifying carbon cycling in CES using pressure sensors. CES of volume 20 mL are housed in glass vials (40 mL total volume), stirred at 450 rpm, illuminated by an LED, and held at 30 ${}^{{}^{\circ}}$C under feedback temperature control (*SI Appendix*). A high-precision pressure sensor is integrated into the hermetically sealed cap and a porous foam stopper (yellow) shades the sensor from illumination. (*D*) Pressure measurements (acquired once per second) in a CES subjected to 12 h–12 h light–dark cycles as indicated by yellow and gray shaded regions, respectively. Light intensity during the light phase is 150 μmol m^– 2^s^– 1^. Pressure rises and falls in response to light and dark as expected. The pressure stabilizes during the light phase, indicating that photosynthesis becomes CO_2_ limited. The change in pressure is proportional to *r* and *f* as labeled. Carbon cycling, computed from these quantities, is proportional to the amplitude of pressure oscillations (*SI Appendix*). Data in *D* are smoothed with a 1-min moving average. A change in pressure of 1.56 hPa (black line, right side) corresponds to a production/consumption of ∼2 μmol of CO_2_ assuming pH 6.5 and photosynthetic/respiratory quotients of 1 (*SI Appendix*).

Carbon cycling arises in CES from the catabolic activity of photosynthetic and heterotrophic microbes. The complementary reactions of oxygenic photosynthesis and aerobic respiration consume (produce) and produce (consume) CO_2_ and O_2_, respectively (Fig. 1*A*). Carbon cycling emerges from the photosynthetic conversion of CO_2_ into organic carbon, which is then either excreted by phototrophic microbes (27) or made available to bacterial decomposers via death of primary producers. The subsequent respiration of organic carbon by bacterial community members produces CO_2_, completing the cycle.

Carbon exchange between microbial phototophs and heterotrophs is important in many ecosystems. For example, in marine microbial communities carbon transfer from autotrophs to heterotrophs is important for the microbial loop (28), which drives ecosystem productivity by coupling photosynthesis to the generation of bacterial biomass and growth at higher trophic levels. In addition, phototroph and bacterial biomass production also has substantial impacts on the uptake of other inorganic nutrients in these ecological contexts (29). High nutrient availability can result in competition for carbon in eutrophic environments, resulting in carbon limiting phototrophic growth (30, 31).

Carbon cycling can be quantified by continuously measuring the production and consumption of O_2_ or CO_2_ in a CES subjected to cycles of light and dark (20). The dependence of photosynthetic O_2_ production (CO_2_ fixation) on light results in oscillations in O_2_ and CO_2_ levels when subjected to diel cycles of light and dark (Fig. 1*B*). We define the carbon cycling rate as the number of moles of carbon cycled (fixed and respired) per light–dark cycle. To quantify carbon cycling, we estimate fixation and respiration rates from measurements of O_2_ and CO_2_ dynamics. Since photosynthesis occurs only during the light phase, we measure the rate of respiration during the dark phase (*r*, Fig. 1 *B* and *D*). We assume that the respiration rate during the light phase is the same as that during the dark phase. Although this assumption can break down in some cases (32), our data show that the respiration rate is stable during the dark phase (*SI Appendix*, Fig. S6), suggesting that bacterial respiration does not change dramatically between light and dark phases. The amount of CO_2_ fixed during the light phase is computed by measuring the net oxygen production (CO_2_ fixed; *f*, Fig. 1*B* and *D*) during the light phase and accounting for the respiration rate to infer a total CO_2_ fixed (*SI Appendix*). The amount of carbon cycled over a light–dark cycle is then the number of moles of inorganic carbon both fixed and produced. Assuming constant photosynthetic and respiratory quotients (ratio of O_2_ production [consumption] to CO_2_ consumption [production]) allows carbon cycling to be quantified by measuring either O_2_ or CO_2_ dynamics under cycles of light and dark (33).

As noted by Obenhuber and Folsome (19), O_2_ has 30-fold lower solubility in water than CO_2_. As a result, when photosynthesis converts water-soluble CO_2_ to lower-solubility O_2_ in a sealed vessel, most of the O_2_ leaves the liquid and goes into the gas phase, increasing the pressure in the head space. Similarly, if O_2_ is consumed by respiration, this reduces the pressure in the head space of the vessel by converting the lower-solubility O_2_ into higher-solubility CO_2_. Therefore, O_2_ dynamics in a sealed vessel can be quantified by simply measuring changes in pressure in the head space under cycles of light and dark (Fig. 1*B*). These changes in pressure can be used to quantify rates of photosynthesis and respiration in situ.

We developed a custom culture device to precisely measure changes in pressure in a CES subjected to cycles of light and dark. A schematic is shown in Fig. 1*C*. Each device housed a 20-mL CES in a 40-mL glass vial. The cap of the hermetically sealed vial was fitted with a high-precision, low-cost, pressure sensor developed for mobile devices (Bosch; BME280). In contrast to direct detection of O_2_ or CO_2_, pressure measurements are higher sensitivity, lower cost, require no calibration, do not consume analyte, and are stable for months. The vial was illuminated from below by a light-emitting diode (LED) and fitted in a metal block that was held under feedback temperature control via a thermoelectric heating–cooling element (34). Thus, our custom culture devices permit real-time quantification of carbon cycling rates in many replicate CES while precisely controlling temperature and illumination. When we subjected the CES housed in our devices to cycles of light and dark (12 h–12 h), we observed increases and decreases in pressure, driven by the production and consumption of oxygen by photosynthesis and respiration during light and dark phases, respectively (Fig. 1*D*). Performing the same experiment with only water in the vial resulted in no pressure oscillations as expected (*SI Appendix*, Fig. S1), and concurrent measurements of O_2_ and pressure in the vial confirmed that pressure changes reflected the production and consumption of O_2_ and therefore CO_2_ (*SI Appendix*, Figs. S2 and S3).

In addition, we considered the possibility that other gases (nitrogen, hydrogen, sulfide) might be produced and consumed by the microbial community, driving changes in pressure. Based on the availability of these compounds in our CES, and the metabolic capabilities of the taxa detected via sequencing (*Top–Down Assembly of Closed Microbial Communities*, *SI Appendix*, and Dataset S6), we concluded that the production and consumption of other gases are not dominating the pressure changes we observe. Consistent with this conclusion, we observe a correlation between the total oxygen produced in our CES and the autotroph relative abundance (*SI Appendix*, Fig. S14). Therefore, the respiration rate (*r*) and net photosynthesis (*f*) can be quantified directly from continuous pressure measurements (Fig. 1*D*). The rate of carbon cycling in our CES is proportional to the amplitude of the light-driven pressure oscillations (*SI Appendix*). However, the conversion factor from pressure measurements to carbon cycling rates depends on the pH of the media and the photosynthetic and respiratory quotients (*SI Appendix*, Eq. **S14**). Since these quantities cannot be measured in situ, the inferred carbon cycling rates have a systematic uncertainty of ∼30% (*SI Appendix*, Fig. S3).

**Fig. 2:**
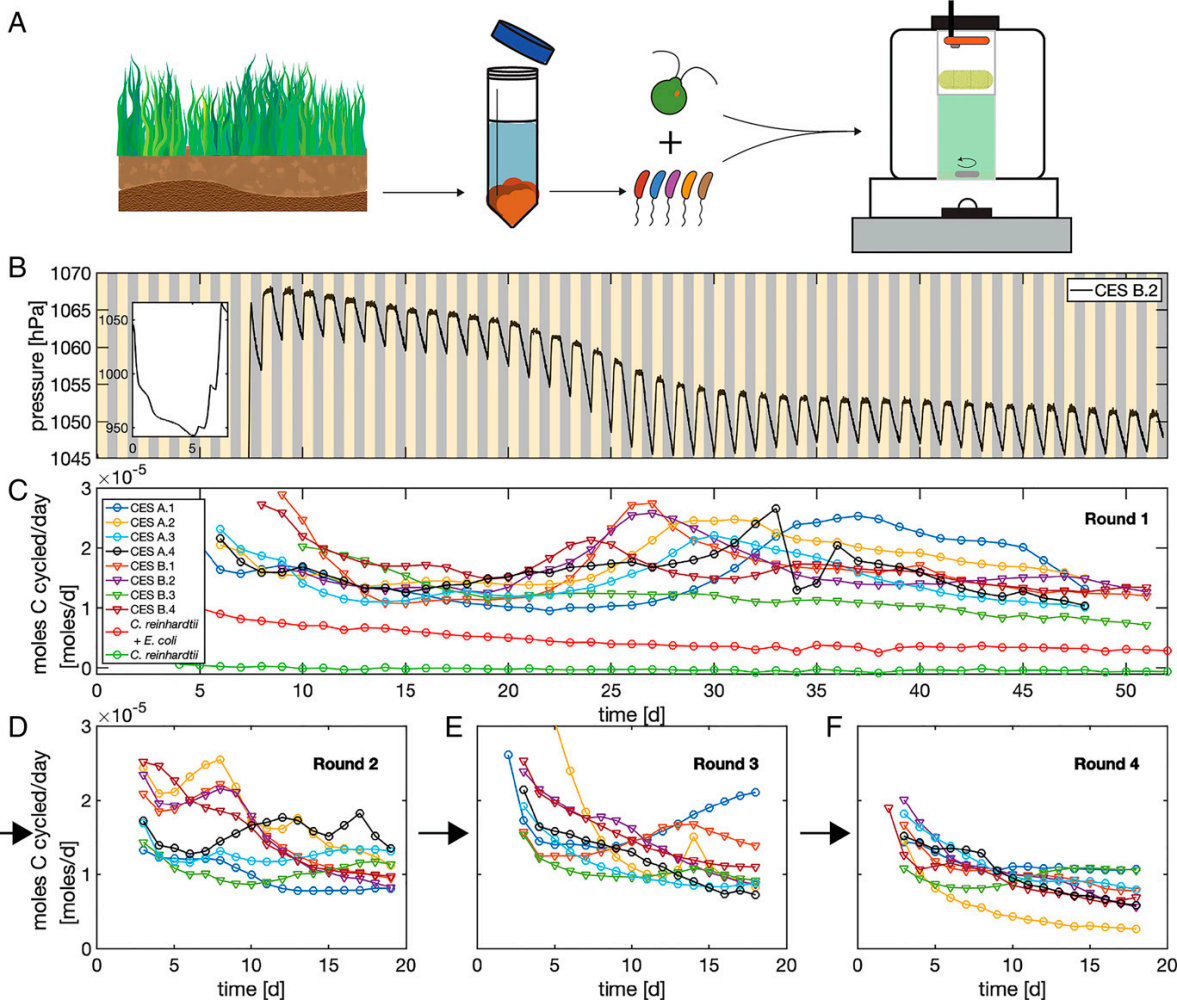
Long-term carbon cycling in closed ecosystems composed of *C. reinhardtii* and soil-derived bacterial communities. (*A*) Top–down assembly of microbial CES. Soil samples are harvested and bacterial communities are extracted. Bacteria are then combined with the alga *C. reinhardtii* and inoculated into the custom culture devices described in Fig. 1*C*. Eight CES were assembled, four each from two soil samples (“A” and “B”), in defined minimal medium, and subjected to 12 h–12 h light–dark cycles (yellow/gray shaded regions) for ∼50 d while pressure was measured. Light intensity was 150 μmol m^– 2^s^– 1^ during the light phase. (*B*) Pressure measurements performed once per second, smoothed by a 1-min moving average, for one of the eight CES. The initial large drop in pressure due to rapid respiration of supplied organic carbon (glucose) is shown in *Inset*. (*C*) The rate of carbon cycling (moles per day) for all eight CES is computed from pressure traces as described in *SI Appendix*. Carbon cycling rates are reported only after the initial transient phase (*B*, *Inset*) has ended. We assume respiratory and photosynthetic quotients of 1 and pH 6.5. Circles indicate CES from soil sample A and triangles those from soil sample B. The transient increase in cycling around 25 to 35 d coincides with a reduction in photosynthetic rates and an increase in respiration (*SI Appendix*, Fig. S7). Red and green traces are synthetic CES composed of *C. reinhardtii* and *E. coli* (mean of two replicates) and *C. reinhardtii* (single replicate; *SI Appendix*, Fig. S4) as shown in the key. Statistical errors in estimates of carbon cycling are smaller than the size of the markers. Key in *C* applies to *D–F*. At the end of the acquisition shown in *C* all eight CES were opened, samples were taken, and CES were diluted 1:20 into fresh media. CES were then sealed for an additional ∼18 d of light–dark cycles and carbon cycling was monitored. (*D*) Carbon cycling rates after the first dilution. Two additional dilution rounds were performed and cycling rates are shown in *E* and *F* as indicated by the black arrows. The average cycling rates at the end of each round do not differ significantly between rounds of enrichment (*P* values: 0.31, 0.87, and 0.053, two-sample *t* test between last measurement between rounds 1 and 2, 2 and 3, and 3 and 4, respectively).

Using these devices, we initially measured carbon cycling in variants of a previously studied synthetic CES (25, 26) composed of *Chlamydomonas reinhardtii* (UTEX 2244, mt+) and *Escherichia coli* (MG1655) over periods of several weeks. We found that the algae alone or algae with *E. coli* failed to persistently cycle carbon (Fig. 2*C* and *SI Appendix*, Fig. S4). We speculate that this failure arose from the production of starch by the algae (35), which cannot be utilized by *E. coli*. Therefore, we reasoned that increasing the metabolic diversity of the bacterial component of our CES might improve carbon cycling. To accomplish this, we turned to a top–down community assembly approach (9, 11) outlined in Fig. 2*A*.

## Top–Down Assembly of Closed Microbial Communities

To assemble communities, we sampled local soils, removed eukaryotes by applying drugs, and extracted bacterial communities using standard techniques (*SI Appendix*). We then combined these diverse bacterial populations with the domesticated soil-dwelling alga *C. reinhardtii* (Fig. 2*A*). We used soil communities to initialize our CES for two reasons. First, since *C. reinhardtii* is native to soil, we reasoned that bacterial communities in soils might more fully recycle nutrients and resources produced by *C. reinhardtii*. Second, soils harbor substantial metabolic diversity (36) and we reasoned that higher-diversity starting communities would be more likely to form stable nutrient cycles in a CES. The resulting CES contained a diverse assemblage of bacteria and the alga. While we were unable to completely exclude photosynthetic bacteria from the soils, our sequencing measurements indicated that the alga dominated the photosynthetic component of our communities (*SI Appendix*, Fig. S13). We assembled eight CES using this method, four each from two soil samples (designated “A” and “B”), and inoculated them into a chemically defined freshwater mimic medium (37) that included organic carbon (glucose), nitrogen (ammonia), and phosphorous (phosphate; *SI Appendix*, Table S4) to facilitate the initial growth of the community. We sealed these communities in vials and placed them in culture devices like the one shown in Fig. 1*C* and subjected them to 12 h–12 h light–dark cycles for a period of ∼50 d.

A representative time series of pressure for one of these CES is shown in Fig. 2*B*. We observed an initial large decline in pressure (Fig. 2 *B*, *Inset*), which arose from the rapid bacterial respiration of glucose (this decline is not present in CES of algae alone; *SI Appendix*, Fig. S4). The pressure remains ∼10% below ambient for 5 to 8 d and then begins to rise (*SI Appendix*, Fig. S5), reflecting the timescale over which we expect algae to grow (38). The rising pressure reflects photosynthetic activity (O_2_ production) by the algae before saturating after 8 to 10 d (*SI Appendix*, Figs. S2 and S5).

Once the pressure saturated, we observed stable pressure oscillations driven by light–dark cycles. In this regime, during each light phase, the pressure stabilized within 2 to 3 h of the illumination being turned on. Therefore, the autotrophs rapidly fix CO_2_ early in the light phase before exhausting the inorganic carbon supply later in the light phase. After CO_2_ is depleted during the early periods of the light phase, respiration and photosynthesis rates are balanced, resulting in stable pressure (O_2_ levels) late in the light phase. We infer that the respiration is the rate-limiting step in the carbon cycle in our CES and that light is not limiting carbon fixation. During the dark phases of each light–dark cycle, we observe a linear decrease in pressure with time, indicating a constant rate of respiration during the dark phase (*SI Appendix*, Fig. S6).

We observed stable pressure oscillations, with saturating pressure levels during the light phase and constant respiration rates during the dark phase, for a period of ∼50 d. During this period, we observe longer-timescale dynamics whereby the pressure (O_2_) levels slowly drop after about 25 d (seven of eight CES; Fig. 2*B* and *SI Appendix*, Fig. S5). A detailed analysis of the O_2_ dynamics reveals that this transient decline in pressure coincides with a slowing of the photosynthetic rates and an increase in the respiration rates (*SI Appendix*, Fig. S7). We speculate that this results from the death of a fraction of the algal population that supplies the bacterial community with additional organic carbon for respiration. After this transient decline, the photosynthetic rate stabilizes (*SI Appendix*, Fig. S7), indicating that a stable population of autotrophs is fixing carbon.

We estimated the rate of carbon cycling in each of our eight CES directly from pressure measurements, like the one shown in Fig. 2*B*, and the results are shown in Fig. 2*C*. We observe robust carbon cycling at rates of ∼10 to nearly 30 μmol Cd^– 1^ in all eight CES. The magnitude of this carbon cycling rate is a sizable fraction of the total organic carbon supplied to each CES at the outset (∼200 μmol; *SI Appendix*, Table S5) and the amount of nonvolatile organic carbon present in each CES at the end of the experiment (120 to 180 μmol; *SI Appendix*, Fig. S8). Therefore, in a period of between 4 and 20 d the amount of carbon cycled approaches the total carbon in the CES. In this sense, we conclude that the carbon cycling rate in our self-assembled CES is high. In contrast, in CES composed of *C. reinhardtii* or *C. reinhardtii* and *E. coli* we observe carbon cycling rates that are below our detection limit and approximately fourfold lower than those of the complex CES, respectively (Fig. 2*C*, green and red circles). We conclude that CES composed of *C. reinhardtii* and complex soil-derived bacterial communities self-organize to rapidly cycle carbon.

A separate control experiment tested the role of algae and light on carbon cycling. CES maintained in the dark exhibited a monotonic decline in pressure, consistent with no photosynthetic activity and no carbon cycling (*SI Appendix*, Fig. S25). CES assembled from soils without *C. reinhardtii* also cycled carbon when provided with light. Sequencing and chlorophyll fluorescence measurements of these CES revealed that carbon cycling was enabled by the presence of bacterial phototrophs native to the soil (*SI Appendix*, Fig. S27 and Dataset S5). However, CES derived from soil alone, without added algae, cycled significantly less carbon than those that included the alga (median 41% decrease; *SI Appendix*, Fig. S25 and section 6.5). By comparison, as shown in Fig. 2, we cannot detect carbon cycling from *C. reinhardtii* alone (<1 μmol d^– 1^). These results indicate that the algae, a diverse bacterial consortia, and illumination are necessary for establishing robust carbon cycling in our CES.

## Taxonomic Characterization of Closed Microbial Communities

How do similar carbon cycling rates across CES emerge from bacterial consortia derived from distinct soil samples? One possibility is taxonomic similarity between assembled bacterial communities. In this scenario, one or a few similar bacterial taxa would rise to high abundance potentially due to their ability to utilize organic carbon produced by *C. reinhardtii* (27) or carbon liberated by algal death. Another possibility is that taxonomically distinct consortia are maintained in each CES despite the similar carbon cycling rates, and it is the metabolic capabilities of the assembled bacterial communities that are similar from one CES to the next and not the taxa present. The latter outcome could arise from functionally redundant bacterial communities (11, 39) that are able to consume the organic carbon produced by *C. reinhardtii* and the lower-abundance bacterial autotrophs but are composed of taxonomically distinct bacteria.

To test between these possibilities we performed an enrichment experiment that allowed us to quantify the taxonomic composition and metabolic properties of our CES, while enriching communities for those taxa essential for carbon cycling. Each CES was opened, sampled, assayed, and diluted 1:20 into fresh medium over three rounds. We chose three rounds of 1:20 dilution to reduce the abundance of any strains not able to grow in our CES by 8,000-fold, putting them below our detection limit by sequencing. The enrichment process also allowed us to harvest sufficient biomass from each CES after each round to perform multiple assays of metabolic activity and taxonomic structure. The enrichment was performed three times (rounds 2 to 4) with ∼18-d periods of closure for each round. Carbon cycling rates during each of these enrichment phases are shown in Fig. 2 *D* and *E* (*SI Appendix*, Fig. S9). In most CES, we observed a decline in carbon cycling rates during the first ∼10 d of closure before rates stabilize across most CES. We found that the average cycling rates at the end of each round of dilution do not differ significantly from one round to the next (Fig. 2). However, one of eight CES exhibited a substantial decline in carbon cycling rates relative to the mean in the final round (CES A.2; yellow curve, Fig. 2*F*). Further, two CES were diluted and sealed again after round 4 and showed stable or increasing cycling rates for an additional period of >200 d (*SI Appendix*, Fig. S10). We conclude that the carbon cycling is robust to serial dilution and that our CES can stably cycle carbon for many months.

**Fig. 3:**
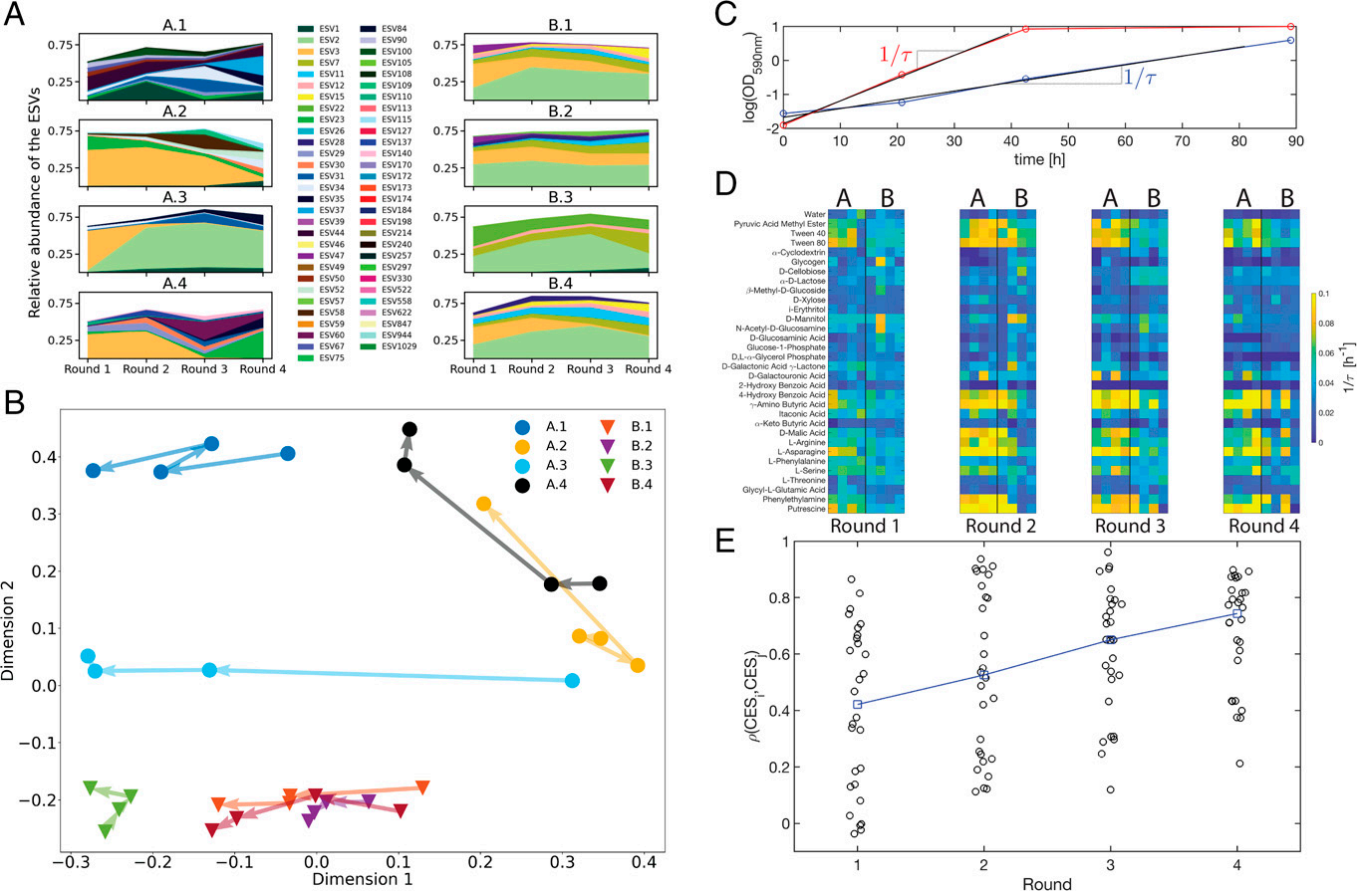
Divergent taxonomic structure and convergent metabolic capabilities across replicate CES. (*A*) Relative abundances measured by 16S rRNA amplicon sequencing of the bacterial taxa comprising the CES (*y* axis) for each round of dilution (*x* axis). Each exact sequence variant (ESV) is represented by a unique color, indicated in the legend. Only the ESVs that have a relative abundance of 5% or higher in at least one of the four dilution rounds for each CES are shown. Most ESVs belong to unique genera (*SI Appendix*, Fig. S21, where multiple ESVs having the same genus are combined). (*B*) The JSD of the relative abundances of all detected taxa at the ESV level is computed between all 32 CES, as described in *SI Appendix*. MDS is applied to the JSD to embed the data in two dimensions. The circles denote CES derived from soil sample A and the triangles denote CES derived from soil sample B; colors correspond to Fig. 2*C*. The arrows indicate transitions between dilution rounds. *SI Appendix*, Figs. S16–S18 show similar results with other metrics. (*C–E*) Measurements of carbon utilization profiles for all eight CES at all four rounds of dilution. (*C*) Two time series of absorbance (590 nm), indicating respiration in Biolog EcoPlates via the redox-sensitive dye tetrazolium ([41]). For each time series, we compute a timescale 1/τ by regressing $\log(OD_{590\;\text{nm}})$ versus time (black traces). We restrict the fit to a time interval between the minimum observed $\log(OD_{590\;\text{nm}})$ and 90% of the maximum $\log(OD_{590\;\text{nm}})$. For carbon sources respired quickly (red), 1/τ is small and the converse. After each dilution round, we measured 1/τ for 32 carbon sources, each in triplicate. In *D* and *E* we consider the quantity 1/τ across CES and dilution rounds. (*D*) The carbon respiration profiles of the eight CES are shown here for each dilution round, with carbon sources in rows and CES in columns. Dilution rounds are shown in separate panels (left to right) as labeled below. In each panel, CES from soil sample A are shown on the left and those from soil sample B on the right. Each entry indicates a mean 1/τ across the three replicate measurements for each carbon source in each CES. (*E*) Correlation between CES carbon utilization profiles in each round of dilution. The Pearson correlation coefficient is computed between each of the distinct columns in *D* (black circles). The median correlation of carbon utilization profiles between CES increases through four rounds (blue). The increase in the median correlation is significant between rounds 1 and 3 and 1 and 4 (*P* values: 0.03, 0.004, respectively, by bootstrapping), but not between rounds 1 and 2 (*P* value: 0.27).

Between each of the four rounds of enrichment (Fig. 2 *C–F*) samples were taken from each of the eight CES. On each of these samples, we performed 16S amplicon sequencing (V4 hypervariable region) of bacterial communities. Fig. 3*A* shows a time series of the dominant taxa in all eight CES across all four rounds of dilution (dominant taxa are those at relative abundance above 5% in any time point). Taxonomically, assembled CES comprise approximately five taxa that make up approximately three-quarters of the population in each community. Some CES exhibit relatively large taxonomic variation from round to round (A.1, A.2, and A.4), while in others we find that the taxonomic structure is relatively stable from round to round (A.2, B.2, B.3; Fig. 3*A*). While all CES from soil sample B harbor a taxon from the genus *Terrimonas*, the same taxon is observed only in a later round of enrichment in one of the four CES from sample A. Further, all CES retain between 80 and 220 rare taxa (relative abundances <5%) with the number declining after round 1 (*SI Appendix*, Fig. S12). Therefore, a visual inspection of Fig. 3*A* suggests that there is no obviously conserved taxonomic structure across our CES.

Further, we find that the bacterial communities in our CES differ strongly from the initial soil samples in terms of composition (*SI Appendix*, Figs. S11 and S28), but not in terms of *α*-diversity (*SI Appendix*, Fig. S22). This, and the results of the control experiments with and without illumination and added algae, suggests that illumination and the presence of algae result in a reorganization of the soil community (*SI Appendix*, Fig. S26*C*).

To quantify taxonomic variability across CES, we computed the Jensen–Shannon divergence (JSD) (40) between the relative abundances in each pair of CES at each round of enrichment. The JSD quantifies differences in community composition between two communities and varies between 0 for two identical communities and 1 for two communities that share no taxa in common. On average, the taxonomic composition differs more between CES (inter-CES) than it does for the same CES across rounds of enrichment (intra-CES; *SI Appendix*, Fig. S15), a result that is robust to using other community similarity metrics (*SI Appendix*, Figs. S16–S18). We also found that the JSD between CES from different soil samples did not decline across rounds of enrichment (*SI Appendix*, Fig. S19), indicating that the taxonomic differences between CES from different soil samples are retained through the enrichment process. Inter-CES divergences remained larger than intra-CES divergences even when we grouped taxa with only 90% 16S sequence similarity, indicating that there is no taxonomic similarity between CES even at higher levels of classification (*SI Appendix*, Figs. S20 and S21). To visualize community taxonomic composition, we embedded the JSD between all CES at all rounds into two dimensions using multidimensional scaling (MDS) (see *SI Appendix*, Fig. S24 for the stress of this embedding) and the result is shown in Fig. 3*B*. Note that the points corresponding to each instance of a CES remain largely separated from each other. Fig. 3*B* supports our assertion that the taxonomic composition differs strongly from one CES to the next and that during enrichment these differences are retained. The differences between CES from soil sample A are larger than those from sample B (*SI Appendix*, Fig. S15), but in neither case did we observe CES converging to a shared taxonomic makeup of the bacterial community. We conclude that the bacterial communities in our CES differ substantially in their taxonomic composition.

## Metabolic Characterization of Closed Microbial Communities

The result that the taxonomic structure differs strongly from one CES to the next despite similar carbon cycling rates supports the idea that carbon cycling in our CES is accomplished by diverse but functionally redundant bacterial communities. Functional redundancy, where different community compositions drive similar metabolic function, is often observed in microbial communities (11, 39). Our observation of functional redundancy suggests that the metabolic capabilities of the assembled bacterial communities might be conserved across CES. Reasoning that the identity of the organic carbon compounds produced by *C. reinhardtii* (and the minor native autotrophs present from the soils) is likely similar across CES, we hypothesized that the carbon utilization capabilities of the assembled bacterial communities might be similar across CES.

To test this hypothesis we measured carbon utilization capabilities on diverse carbon sources for all CES after each round of enrichment. To accomplish this we used Biolog 96-well EcoPlates (41) that exploit a redox-sensitive dye to report respiration in the presence of 32 diverse carbon sources (including compounds excreted by *C. reinhardtii*; *SI Appendix*, Table S6), each in triplicate. After each round of dilution we distributed aliquots of each CES into an EcoPlate. We then incubated the plates and measured dye absorbance, a proxy for carbon respiration, daily for a period of 4 d. Example absorbance time series are shown in Fig. 3*C*. For each replicate of each carbon source, we computed a rate of respiration for that carbon source (1/τ) by estimating $\Delta \log(OD_{590})/\Delta T$, where *OD*_590_ is the absorbance signal reporting respiration (Fig. 3*C* and *SI Appendix*). The quantity 1/τ approximates the rate at which a CES utilizes a given carbon compound. We averaged 1/τ across the three replicates for each carbon source at each round of enrichment in each CES (Fig. 3*D*). Each row of Fig. 3*D* shows the average 1/τ (utilization rate) for a single carbon source and each column a profile for a CES. Comparing carbon utilization profiles across rounds reveals a convergence in the metabolic capabilities across our eight CES, with profiles becoming more similar across CES as the number of rounds of enrichment increases. For example, by the end of round 4 none of the CES utilize 2-hydroxy benzoic acid despite six of eight CES being capable of consuming the carbon source after round 1. Conversely, the enrichment process increases 1/τ for other carbon sources (phenylethylamine, putrecine, *γ*-amino butyric acid). We note that the carbon utilization profiles of the enriched CES, after round 4, differ strongly from those of *E. coli* (*SI Appendix*, Fig. S29), which itself fails to cycle carbon with *C. reinhardtii* (Fig. 2*C*), suggesting that the carbon utilization capabilities of the complex CES are important for stable carbon cycling.

To quantify the variation in the carbon utilization profiles across CES we computed the cross-correlation coefficient in carbon utilization between every pair of CES in each round (columns, Fig. 3*D*). The results are shown in Fig. 3*E* where we observe a steady and statistically significant increase from round 1 to round 4. This correlation measures the similarity between pairs of CES in their carbon utilization profiles, and thus the increase we observe quantifies the extent to which CES are converging over rounds of enrichment to a similar carbon utilization profile.

We speculated that the metabolic convergence we observe in Fig. 3*E* might be a consequence of carbon limitation in our CES. For example, if organic carbon is limiting and provided to bacteria by the algae either by excretion or by cell death, then the spectrum of carbon compounds provided by the algae would determine the carbon catabolic capabilities that the bacterial community must possess to utilize the available carbon. Indeed, a control experiment indicates that some of the compounds utilized by the assembled bacterial communities are excreted by *C. reinhardtii* (*SI Appendix*, Table S6 and Dataset S3). However, from the pressure data or metabolic profiling, we cannot determine the nutrient-limiting respiration in our CES. To address this question we performed an assay after each round of dilution to determine the nutrient-limiting respiration. We used a Microresp assay (*SI Appendix*) whereby small aliquots of each CES were dispensed into 96-well plates and supplemented with carbon, nitrogen, or phosphorous. We measured CO_2_ production in each sealed well directly using a pH-sensitive dye and compared the results to control wells where no nutrients were added (*SI Appendix*, Fig. S30). We found respiration in our CES was in some cases carbon limited, in some cases phosphorous limited (predominantly in CES from soil sample A; *SI Appendix*, Fig. S30), or in some cases both (community A.1). A quantitative analysis of the nutrient budgets in our CES and literature values for the stoichiometry of biomass revealed that, given the excess phosphorous in the media, phosphorous limitation most likely arises from phosphate storage, either by bacteria (42) or by *C. reinhardtii* (43) and not the incorporation of phosphorous into new biomass (*SI Appendix*, section 7.2.2). These results suggest that sequestration may change the nutrient-limiting respiration in our CES, but that the metabolic convergence we observe (Fig. 3 *D* and *E*) is robust to limitation by other nutrients. A more complete accounting of the mechanisms governing nutrient limitation in these communities will require a detailed interrogation of respiratory coefficients, biomass stoichiometry, and carbon transfer between the autotroph and bacterial components.

## Discussion

The primary results of our study are the demonstration that CES can be powerful model microbial ecosystems for studying nutrient cycling and the development of a high-resolution method for quantifying cycling in closed communities. Model systems have proved essential for advancing every area of biology, including from gene expression (44), to development (45), to evolution (46). However, we lack model systems to serve the same purpose at the level of the community or ecosystem (18). Since CES are closed, nutrient cycling is required for persistence. Therefore, CES constitute model systems for studying nutrient cycling at the level of the collective with the key property of permitting control of community composition, nutrient, and energy availability. Given this tractability, CES constitute model biospheres for understanding how communities are organized to satisfy the constraints placed on them by nutrient cycling and for learning how evolutionary processes impact this organization. One of the main limitations in the field was a lack of precise, long-term, in situ measurements of nutrient cycling. We have overcome this limitation and demonstrated that CES are amenable to quantitative measurements of nutrient cycling while interrogating community structure at the taxonomic and metabolic levels.

Our taxonomic and metabolic characterization of replicate CES showed that carbon cycling in CES can be sustained by diverse bacterial consortia that exhibit a conserved set of metabolic capabilities. The result points to the idea that the emergent functional property of carbon-cycling microbial communities is likely a conserved set of metabolic capabilities (39) that are robust to variation in the taxonomic structure of the system. However, some aspects of community function that we have not measured may depend on the taxonomic structure of the community, such as phosphorous sequestration. Ultimately, the functional aspects of the community that can be performed by diverse taxa likely depend on the phylogenetic conservation of the associated phenotypic traits. In this context, our data suggest that the conserved properties of carbon-cycling CES are likely carbon utilization pathways and the taxonomic diversity in our CES potentially reflects the weak phylogenetic conservation of carbon utilization phenotypes (47). It will be interesting to extend this study to understand the role of this taxonomic variability and metabolic convergence in determining the robustness of nutrient cycling to environmental perturbations such as changes in temperature or light levels. While previous studies have considered functional robustness in communities (48), our CES offer the advantages of real-time measurements of community function for many replicate consortia in the laboratory.

The fact that CES are hermetically sealed means that they differ markedly from natural communities where immigration can change the makeup of the community. Despite this difference, we propose that CES can act as model systems for understanding how nutrient cycling constrains the structure of a community. While immigrations can and do alter the taxonomic structure of communities in the wild, it is frequently observed that metagenomic structure is tightly coupled to abiotic factors (39), suggesting that the assembly of functional communities may be deterministic given specific environmental contexts (8, 26). In this case, provided a CES is initialized with sufficient metabolic diversity to satisfy the constraints on the system set by cycling, the final functional structure of the community may not depend strongly on whether or not immigrations are allowed to occur, a hypothesis that could be tested by opening CES and introducing invaders.

Nutrient cycling in wild microbial communities often involves recycling of a single nonsubstitutable nutrient such as sulfur (5) or carbon (49), with other essential nutrients available in excess. This is in contrast to CES where no nutrients are supplied exogenously and biomass generation requires cycling all nutrients at once. In our CES it remains unclear to what extent nutrients other than carbon are cycled, such as those primarily involved in anabolism (N, P, Fe). It may be that the generation times in our CES are long, yielding few cell divisions in the course of the experiment. In this case carbon exchange could be utilized by algae and bacteria for maintenance energy. In this situation the cycling of nutrients such as N or P would be slow. In contrast, if generation times are short and many generations occur over the course of an experiment, nutrients such as N and P would need to be rapidly cycled to sustain cell division (50).

In addition, quantifying abundance dynamics and metabolite exchanges in our CES would reveal how specific ecological interactions endow these communities with stable cycling capabilities. Detailed data on abundance dynamics would also permit comparison between our experiments and the substantial existing body of theoretical work on closed ecosystems (51–54). In particular, because the energy available to the system is readily varied by changing light intensity, CES could be used to test the proposed role of energetics in determining community structure (55).

CES have a key role to play in future work understanding evolution at the level of the community. Simulations and directed evolution approaches have been used to ask whether and how ecosystem-level traits can be selected (56, 57). As with directed evolution in proteins or organisms, the target of adaptation by the ecosystem is typically stipulated by the experiment. For example, communities might be selected for the production (58) or degradation (56) of a particular compound. Prior work in this field has faced two problems. First, it has been challenging to perform selection in the laboratory on a community-level trait that cannot be optimized by adaptation of an individual member of the community (57, 59). For example, selecting a community for fast degradation of a compound can result in simply selecting the strain that degrades that compound most rapidly. Second, community-level evolution requires a notion of heritability, whereby successive generations of a community retain emergent traits of the parent community. However, theoretical work suggests a way to circumvent these obstacles: When selection acts on interaction-dependent properties of the ecosystem, such as metabolite exchange between strains, individual traits evolve to improve community heredity (60). Consistent with this expectation is the proposal that communities mediated by competition or exchange of resources can behave as cohesive units exhibiting emergent traits that are transmitted between generations (61). However, experimentally selecting a community on the basis of an emergent function that relies on interactions between constituents is a challenge. Nutrient-cycling closed ecosystems would appear to be ideal systems to address this problem since carbon cycling requires cooperative metabolic processes. Moreover, nutrient cycles in CES are frequently observed to be self-regulating (19). For example, autotroph senescence could provide additional carbon to heterotrophs and thus increase respiration, enhancing autotroph growth, a process that likely occurs in our CES. Self-regulation removes the requirement that the experimenter fine tune parameters to maintain functional stability (58). Therefore, the dual properties of obligate metabolic interactions to ensure persistence and self-regulation suggest that CES would be natural candidates for ecosystem breeding or long-term experimental evolution. Such efforts could yield insights into the coevolutionary dynamics governing symbioses in natural communities and potentially the eco-evolutionary dynamics of genome streamlining (62).

Given these possibilities, we propose that CES, coupled with careful measurements of metabolite dynamics like those made here, constitute powerful model systems for the quantitative study of emergent nutrient cycling in communities.

## Materials and Methods

For a detailed description of experimental methods and data analysis see *SI Appendix*.

### Pressure Measurement and Assembling Communities

Pressure measurements inside the sealed vessel were made using a Bosch BME280 combined pressure, temperature, and humidity sensor purchased from Amazon. Sensors were integrated into metal caps using a hermetically sealing epoxy. Sensor readout, temperature feedback, and control of illumination were performed by a Raspberry Pi computer running custom Python scripts. Closed ecosystems were assembled from soil samples harvested from a restored prairie near Urbana, IL. Eukaryotes were removed from soil samples with drugs and samples were incubated in the dark for 2 d to minimize the growth of native phototrophs. Soil communities were then combined with *C. reinhardtii* in a chemically defined freshwater mimic medium and sealed.

### Sequencing and Metabolite Analyses

Sequencing was performed using a MiSeq Illumina platform and the Earth Microbiome 16S amplicon sequencing protocol. Data were analyzed using QIIME and DADA2 pipelines. Measurements of carbon utilization were performed with the Biolog Ecoplate following the manufacturer guidelines. Plates were incubated in a humidified bag to avoid evaporation. To measure nutrients limiting respiration, samples of CES amended with nutrients (C, N, P) were placed in deep 96-well plates. An indicator plate, containing agar and a pH-sensitive dye, was hermetically sealed to the deep-well plate and incubated for 24 h in the dark. The indicator plate was removed and absorbance was measured via a plate reader. A calibration experiment quantified CO_2_ production from a change in pH.

## Data Availability

All study data are included in this article and/or supporting information. Additional data are available in Illinois Data Bank (https://doi.org/10.13012/B2IDB-8967648_V1).
